# Functional Dissection of the Proton Pumping Modules of Mitochondrial Complex I

**DOI:** 10.1371/journal.pbio.1001128

**Published:** 2011-08-23

**Authors:** Stefan Dröse, Stephanie Krack, Lucie Sokolova, Klaus Zwicker, Hans-Dieter Barth, Nina Morgner, Heinrich Heide, Mirco Steger, Esther Nübel, Volker Zickermann, Stefan Kerscher, Bernhard Brutschy, Michael Radermacher, Ulrich Brandt

**Affiliations:** 1 Molecular Bioenergetics Group, Medical School, Cluster of Excellence Frankfurt “Macromolecular Complexes,” Center for Membrane Proteomics, Johann Wolfgang Goethe-Universität, Frankfurt, Germany; 2 Institute of Physical and Theoretical Chemistry, Cluster of Excellence Frankfurt “Macromolecular Complexes,” Centre for Membrane Proteomics, Johann Wolfgang Goethe-Universität, Frankfurt, Germany; 3 University of Vermont, College of Medicine, Department of Molecular Physiology and Biophysics, Burlington, Vermont, United States of America; Buck Institute, United States of America

## Abstract

Mitochondrial complex I, the largest and most complicated proton pump of the respiratory chain, links the electron transfer from NADH to ubiquinone to the pumping of four protons from the matrix into the intermembrane space. In humans, defects in complex I are involved in a wide range of degenerative disorders. Recent progress in the X-ray structural analysis of prokaryotic and eukaryotic complex I confirmed that the redox reactions are confined entirely to the hydrophilic peripheral arm of the L-shaped molecule and take place at a remarkable distance from the membrane domain. While this clearly implies that the proton pumping within the membrane arm of complex I is driven indirectly via long-range conformational coupling, the molecular mechanism and the number, identity, and localization of the pump-sites remains unclear. Here, we report that upon deletion of the gene for a small accessory subunit of the *Yarrowia* complex I, a stable subcomplex (*nb8m*Δ) is formed that lacks the distal part of the membrane domain as revealed by single particle analysis. The analysis of the subunit composition of holo and subcomplex by three complementary proteomic approaches revealed that two (ND4 and ND5) of the three subunits with homology to bacterial Mrp-type Na^+^/H^+^ antiporters that have been discussed as prime candidates for harbouring the proton pumps were missing in *nb8m*Δ. Nevertheless, *nb8m*Δ still pumps protons at half the stoichiometry of the complete enzyme. Our results provide evidence that the membrane arm of complex I harbours two functionally distinct pump modules that are connected in series by the long helical transmission element recently identified by X-ray structural analysis.

## Abstract 2

A catalytically active subcomplex of respiratory chain complex I lacks 14 of its 42 subunits yet retains half of its proton-pumping capacity, indicating that its membrane arm has two pump modules.

## Author Summary

Mitochondria—the power plants of eukaryotic cells—produce energy in the form of ATP. More than one-third of this energy production is driven by a gradient of protons across the mitochondrial membrane created by the pumping action of a very large enzyme called complex I. Defects in complex I are implicated in numerous pathological processes like neurodegeneration and biological aging. Recent X-ray structural analyses revealed that complex I is an L-shaped molecule with one arm integrated into the membrane and the other sticking into the aqueous interior of the mitochondrion; the chemical reactions of the enzyme take place in this hydrophilic arm, clearly separated from proton pumping that must occur somewhere in the membrane arm. To assign the pump function to structural domains, we created a stable subcomplex of complex I by deleting the gene encoding one of its small subunits in a yeast called *Yarrowia lipolytica*. This subcomplex lacked half of the membrane arm; it was still catalytically active but it pumped only half the number of protons as the full complex. This indicates that complex I has two functionally distinct pump modules operating in its membrane arm.

## Introduction

Respiratory complex I (proton-pumping NADH:ubiquinone oxidoreductase) is a very large membrane integral multiprotein complex found in most energy converting membranes of bacteria and eukaryotes [1]. It plays a central role in cellular energy metabolism, and complex I defects have been implicated in a number of bioenergetic diseases including major neurodegenerative disorders [2] and biological aging [3]. The enzyme of the inner mitochondrial membrane contains one FMN and eight iron-sulfur clusters as redox prosthetic groups and is composed of >40 different subunits with a total molecular mass of almost 1,000 kDa. Fourteen “central” subunits are conserved among pro- and eukaryotes and form the functional core of complex I. Little is known about the function of the remaining >26 “accessory” subunits [1]. Mitochondrial complex I links the electron transfer from NADH to ubiquinone to the pumping of four protons from the matrix into the intermembrane space [4]. Recent progress in the X-ray structural analysis of prokaryotic [5] and eukaryotic [6] complex I confirmed that the redox reactions are confined entirely to the hydrophilic peripheral arm [7] of the L-shaped molecule [8] and take place at a remarkable distance from the membrane domain [9],[10]. While this clearly implies that the proton pumping within the membrane arm of complex I is driven indirectly via long-range conformational coupling, the molecular mechanism and the number, identity, and localization of the pump-sites remain unclear.

The 180 Å long membrane arm or P module of complex I is subdivided into two subdomains of approximately equal size, called the proximal or P_P_ and the distal or P_D_ module [6]. Structural analysis revealed a very long helix running in parallel to the membrane that has been implicated as a mechanical transmission element exerting conformational coupling between these two subdomains [5],[6]. Based on their homology to bacterial Mrp-type Na^+^/H^+^ antiporters, the three largest subunits of the membrane arm ND2, ND4, and ND5 have been discussed as prime candidates for harbouring the proton pumps [11]. Indeed subunits ND4 and ND5 form the core of the P_D_ module, suggesting that this part of complex I located well over 100 Å apart from the closest redox-center contributes to proton pumping. However, it cannot be concluded from homology alone that the Mrp-type subunits are functional in complex I. A recent mutagenesis study of subunit ND5 in *Escherichia coli* showed the functional importance of this protein for redox-linked proton pumping [12], but otherwise the contribution of the different parts of the membrane arm of complex I to proton pumping and the number of functional pumping modules has not been addressed experimentally. Here, we report that a subcomplex lacking specifically the P_D_ module of the membrane arm is still capable of pumping protons at half the stoichiometry of holo-complex I.

## Results and Discussion

### The Deletion of Subunit NB8M Results in a Stable Subcomplex Lacking Specifically the P_D_ Module

When we deleted the gene for the accessory subunit NB8M from the genome of the strictly aerobic yeast *Yarrowia lipolytica* by homologous recombination, a defined subcomplex of complex I was found in mitochondria from knock-out strain *nb8m*Δ. Subunit NB8M has a molecular mass of 11.1 kDa [13] and is the homologue of subunit B18 of bovine heart complex I [14]. It has no predicted transmembrane helices and carries a twin CX_9_C motif characteristic for mitochondrial proteins imported via the so called MIA-pathway [15]. It has been proposed that it resides at the intermembrane space side of the P_D_ module of complex I [16]. NADH:hexamineruthenium (HAR) oxidoreductase activity that monitors the amount of functional N-module of complex I of mitochondrial membranes prepared from deletion strain *nb8m*Δ was normal. Also no significant changes in the signatures of the iron-sulfur clusters were observed by electron paramagnetic resonance spectroscopy (unpublished data). In contrast, inhibitor sensitive dNADH:decylubiquinone (DBQ) oxidoreductase activity reflecting the physiological function of complex I was reduced to 30% of that of the parental strain (Table S1).

**Figure 1:**
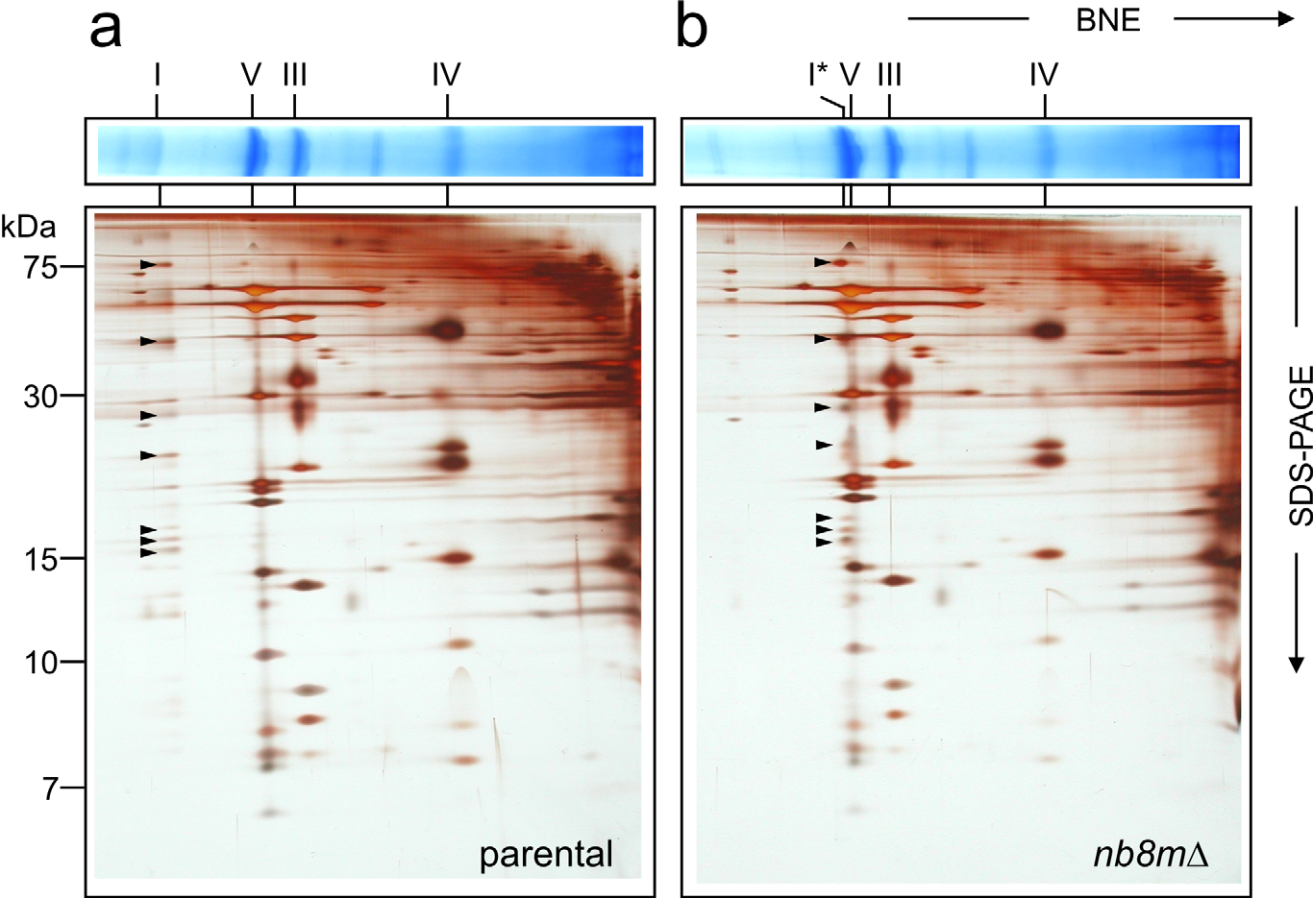
Deletion of subunit nb8m generates a distinct subcomplex. Mitochondrial membranes of parental (a) and *nb8m*Δ deletion (b) strain were separated on blue-native gels (BNE) and subsequently by SDS-PAGE. Positions of respiratory chain complexes (I, V, III, and IV), subcomplex *nb8m*Δ (I*), and complex I subunits (arrows) are indicated.

Further analysis by two-dimensional blue-native polyacrylamide electrophoresis (2D BN/SDS-PAGE) revealed that instead of fully assembled complex I strain *nb8m*Δ contained a defined subcomplex (Figure 1) migrating slightly above complex V (F_1_F_O_-ATP synthase) at an apparent molecular mass of about 600 kDa [17]. The identity of this band as a subcomplex of complex I was confirmed by in-gel activity staining and Western blotting and a very small amount of fully assembled complex I was found to be still present in strain *nb8m*Δ (Figure S1).

**Figure 2:**
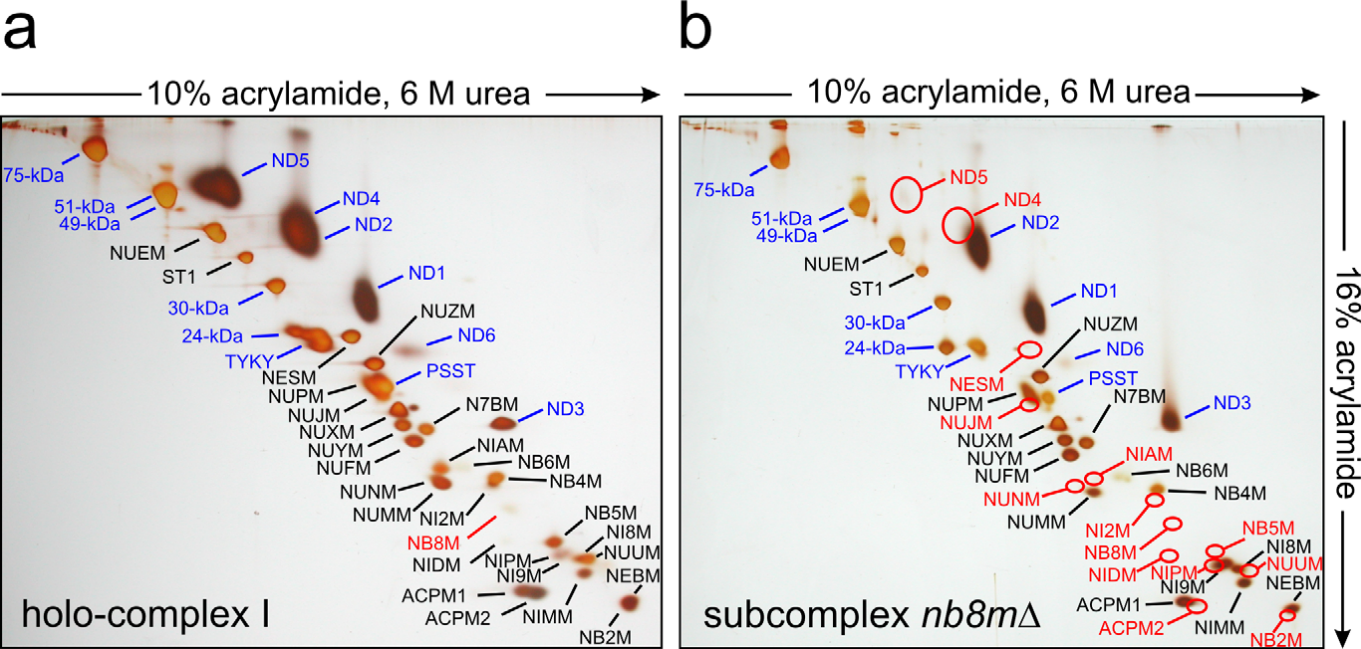
Subunit composition of subcomplex nb8mΔ. Purified complex I (a) and subcomplex *nb8m*Δ (b) were analyzed by doubled-SDS-PAGE. Identified (blue, central; black, accessory) and missing (red, circles) subunits are highlighted (compare Table 1).

Since we had constructed the deletion of the gene for subunit NB8M in a strain expressing the 30-kDa subunit with a his-tag, we could purify the subcomplex using dodecyl-maltoside as a detergent by the same affinity-purification protocol established previously for holo-complex I [18]. The subcomplex eluted as a symmetrical peak from the final size exclusion column and as judged by its specific NADH:HAR oxidoreductase activity was of similar purity (unpublished data). To determine the subunit composition of subcomplex *nb8m*Δ we applied doubled sodium dodecyl sulfate polyacrylamide electrophoresis (dSDS-PAGE; [19]) and laser induced liquid bead ion desorption (LILBID) and electrospray ionisation (ESI) mass spectrometry (MS) as three complementary proteomic approaches that together allowed reliable identification of all subunits present. dSDS-PAGE is especially suited to separate highly hydrophobic proteins that migrate above the diagonal in these gels (Figure 2). LILBID-MS at high laser intensity dissociates non-covalently bound subunits of enzyme complexes and generates a complete mass fingerprint in a single experiment (Figure S2). Most subunits present in the subcomplex were clearly identified by all three methods. In cases where there were weak or overlapping spots in dSDS-PAGE, non-separated peaks in LILBID-MS, or difficulties in identifying specific subunits by ESI-MS, the other methods still allowed to decide whether a subunit was present or not. We found that 14 subunits were missing in subcomplex *nb8m*Δ (Table 1) adding to a total molecular mass of 281,189 Da. Most notably the two mitochondrially coded Na^+^/H^+^ antiporter homologous subunits ND4 and ND5 were absent. As these are the only central subunits previously assigned to the distal portion of the membrane arm [5], this suggested that subcomplex *nb8m*Δ specifically lacked the P_D_ module.

**Table 1:** Subunits of complex I from *Y. lipolytica* indicating proteins missing in subcomplex *nb8m*Δ (bold and underlined) according to combined data from dSDS-PAGE, LILBID-MS, and ESI-MS.

	Subunit	Ortholog *B. taurus*	M_r_ Mature, Da	Module	Identified in Subcomplex *nb8m*Δ by ESI-MS^a^
1	NUAM	75-kDa	75,199	Q/N	yes
2	NUBM	51-kDa	51,660	Q/N	yes
3	NUCM	49-kDa	49,945	Q/N	yes
4	NUGM	30-kDa	30,476^b^	Q/N	yes
5	NUHM	24-kDa	24,069	Q/N	yes
6	NUIM	TYKY	22,321	Q/N	yes
7	NUKM	PSST	20,426	Q/N	yes
8	NU1M	ND1	38,348	P_P_	yes
9	NU2M	ND2	53,332	P_P_	n.d.^c^
10	NU3M	ND3	14,471	P_P_	yes
11	**NU4M**	ND4	54,481	P_D_	no
12	**NU5M**	ND5	73,705	P_D_	no
13	NU6M	ND6	20,758	P_P_	n.d.
14	NULM	ND4L	9,811	P_P_	n.d.
15^d^	NUEM	39-kDa	40,434	Q/N	yes
16	ST1	—	34,490	unknown	n.d.
17	**NESM**	ESSS	23,438	P_D_	no
18	**NUJM**	B14.7	20,696	P_D_	no
19	NUZM	B14.5a	19,750	Q/N	yes
20	NUPM	PGIV	19,196	P_P_	yes
21	NUXM	—	18,565	P_P_	yes
22	N7BM	B17.2	16,153	Q/N	yes
23	NUYM	AQDQ	15,940	Q/N	yes
24	NUFM	B13	15,573	Q/N	yes
25	**NIAM**	ASHI	14,642	P_D_	no
26	NB4M	B14	14,627	P_P_	yes
27	NB6M	B16.6	13,960	P_P_	yes
28	**NUNM**	—	13,301	P_D_	no
29	NUMM	13-kDa	13,117	Q/N	yes
30	**NI2M**	B22	12,749	P_D_	no
31	**NB8M**	B18	11,068	P_D_	no
32	**NIDM**	PDSW	10,890	P_D_	no
33	**NB5M**	B15	10,348	P_D_	no
34	**ACPM2**	—	10,092^e^	P_D_	no
35	**NIPM**	PFFD	9,887	P_D_	no
36	NIMM	MWFE	9,662	P_P_	yes
37	**NUUM** f	—	9,652	P_D_	no
38	ACPM1	SDAP	9,636^e^	P_P_	yes
39	NI8M	B8	9,473	Q/N	yes
40	NI9M	B9	8,981	P_P_	yes
41	NEBM^g^	—	7,917	P_P_	yes
42	**NB2M**	B12	6,805	P_D_	no

a Unpublished data. Purified subcomplex *nb8m*Δ was run on BN-PAGE, subjected to tryptic in-gel digestion and analysed by nano liquid chromatography ESI-MS.

b Molecular mass includes 1,267.3 Da for the hexa-histidine tag and hexa-alanine spacer [18].

c Not determined; the hydrophobic subunits ND2, ND6, and ND4L are not reliably identified by ESI-MS and subunit ST1 is known to dissociate from the complex during BN-PAGE [13].

d Accessory subunits are numbered according to their molecular mass. Not that this numbering is not identical to Morgner et al. [13].

e Molecular mass includes 564.7 Da for covalently bound phosphopantetheine-hydroxy-tetradecanoate.

f Subunit NUUM was recently found in *Y. lipolytica* complex I [35], but we found an intron near the N-terminus resulting in a larger protein that lacked the N-terminal methionine. This was confirmed by peptide identification with LC/ESI-MS analysis at 100% sequence coverage (not shown).

g Subunit NEBM was identified and characterized recently in another study from our laboratory (E. Nübel et al., in preparation).

**Figure 3:**
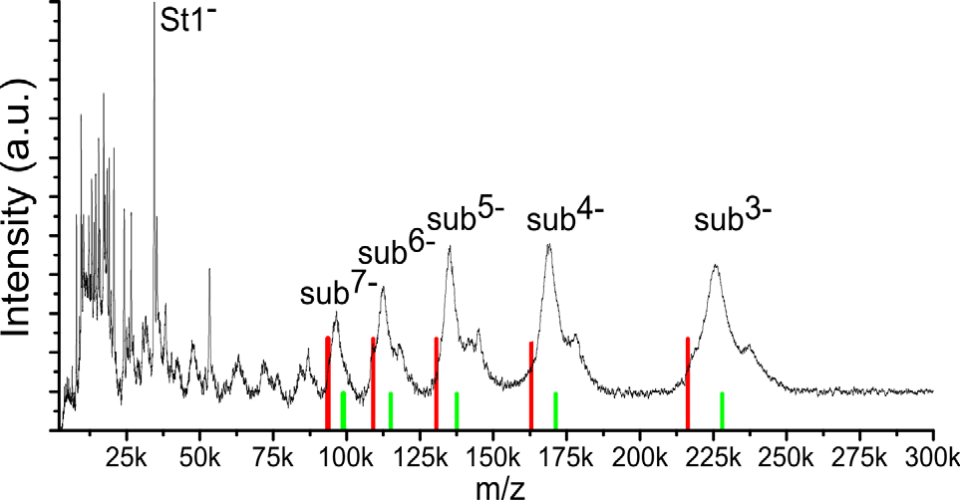
LILBID mass spectra of purified subcomplex nb8mΔ. The predominant series of LILBID-peaks from the subcomplex indicated a protein mass of ∼645 kDa (red ticks). A series of minor peaks (green ticks) indicate that the rather loosely attached subunit ST1 was dissociated during ionisation from the major fraction even under ultrasoft conditions.

To determine the total mass of purified subcomplex *nb8m*Δ, we next applied non-destructive LILBID-MS at ultrasoft conditions. As shown previously, under these conditions the peak maxima indicate the mass of multiprotein membrane protein complexes including detergents and phospholipids that are still bound to the ionized species [13]. Therefore, the lower mass onset of the peaks reflects the contribution of the protein to the total mass. In the case of subcomplex *nb8m*Δ this offset corresponded to ∼30 kDa. Thus the predominant series of LILBID-peaks from the subcomplex indicated a protein mass of ∼645 kDa (Figure 3, red ticks). However, a series of minor peaks showed that, as previously observed for holo-complex I [13], the rather loosely attached subunit ST1 was dissociated during ionisation from a major fraction of the complexes even under ultrasoft conditions. Indeed a prominent peak at m/z 35 kDa matched the single-charged form of this protein (Figure 3). The positions of the maxima of the minor peaks were consistent with the then expected mass of ∼680 kDa for subcomplex *nb8m*Δ (Figure 3, green ticks). This fitted nicely with the proteomic data from which a residual mass of 682.5 kDa was calculated (Table 1).

### 3D Electron Microscopy Structure of Subcomplex *nb8m*Δ

**Figure 4:**
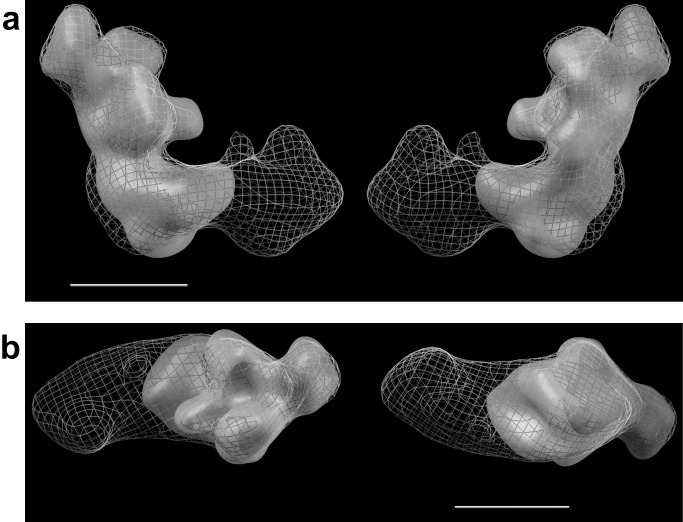
Subcomplex *nb8mΔ* has completely lost the P_D_ module. The 3D electron microscopy structure of subcomplex *nb8m*Δ fitted into the 3D-model of holo-complex I confirmed the absence of the distal part of the membrane arm (P_D_ module). (a) side views; (b) top and bottom views; scale bar 10 nm.

To further investigate the structure of subcomplex *nb8m*Δ, we performed electron microscopic single particle analysis. Figure 4 shows the 3D reconstruction of the subcomplex at a resolution of 23 Å calculated from 10,586 tilt images from a random conical data set with a tilt angle of 55°. We compared the 3D model of subcomplex *nb8m*Δ to that of holo-complex I. The fit of the 3D model of the subcomplex into the outline of the 3D reconstruction of holo-complex I from [9] nicely showed that indeed the P_D_-module of the membrane arm was missing and that the remaining parts exhibited no major structural rearrangements (Figure 4). With the distal end of the membrane arm, also the central and distal membrane arm protuberances were missing, which we had putatively assigned to be in close proximity to the proton translocating subunits ND4 and ND5.

### Subcomplex *nb8m*Δ Pumps Protons at Half the Stoichiometry of the Complete Enzyme

**Figure 5:**
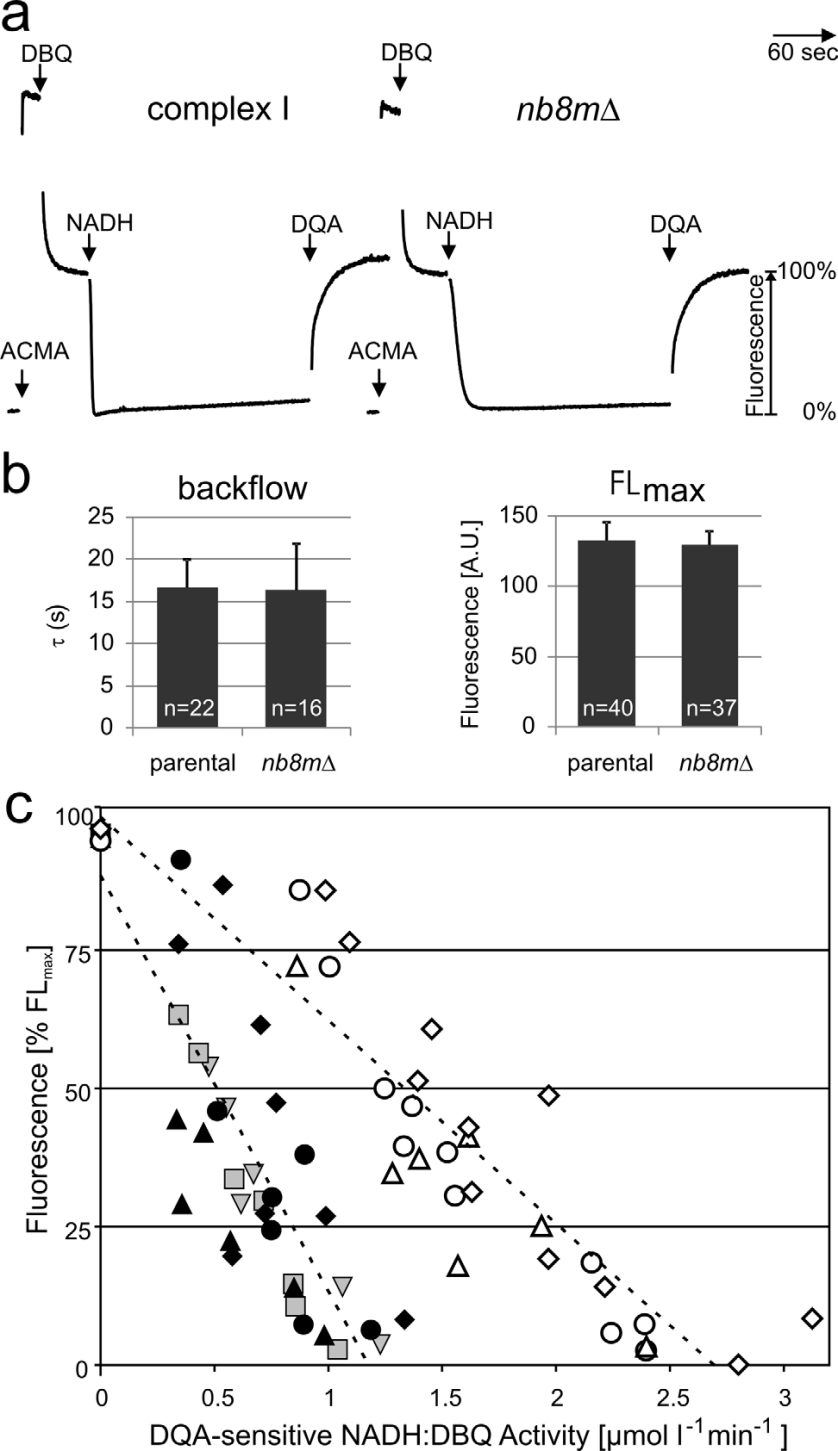
Subcomplex *nb8mΔ* pumps protons with reduced H^+^/e^−^ stoichiometry. (a) Proton pumping of reconstituted complex I and subcomplex *nb8m*Δ monitored by ACMA. Additions of ACMA, substrates (100 µM DBQ, 100 µM NADH), and inhibitor (10 µM DQA) are indicated. (b) The halftime τ for the proton backflow after addition of DQA (left panel) was determined (see Figure S5) and, like the maximal fluorescence FL_max_ (right panel), was found to be essentially the same for complete complex I and subcomplex *nb8mΔ.* (c) The fluorescence at the steady state quench plateau of 5 (filled symbols, holo-complex I) and 3 (open symbols, subcomplex *nb8m*Δ) independent reconstitution experiments were plotted against the corresponding DQA-sensitive NADH:DBQ oxidoreductase activities (Figure S4). The uninhibited electron transport rates in the presence of uncoupler for these preparations were 6.5±0.7 µmol min^−1^ mg^−1^ for the parental strain and 2.5±0.3 µmol min^−1^ mg^−1^ for strain *nb8m*Δ. For parental complex I data for activities greater than ∼1.3 µmol min^−1^ mg^−1^ were omitted since at these high rates ACMA fluorescence was quenched completely. Measurements performed in parallel are marked with symbols of the same shape (e.g., ▴, ▵). The ratio between the slopes for holo-complex I (−75±7%·l·min µmol^−1^) and subcomplex *nb8m*Δ (−36±3%·l·min µmol^−1^) indicated a proton pumping stoichiometry of 2.1 H^+^/2 e^−^ for the subcomplex. When analyses with two additional preparations of subcomplex *nb8m*Δ were included (unpublished data), the overall average stoichiometry for a total of five independent pairs of experiments was calculated as 1.8±0.3 H^+^/2 e^−^.

After lipid-reactivation with asolectin [20] the subcomplex purified from strain *nb8m*Δ exhibited an inhibitor sensitive NADH:DBQ oxidoreductase activity of 2.7 µmol·min^−1^·mg^−1^. At an activity of 5.8 µmol·min^−1^·mg^−1^ for purified holo-complex I and taking into account the lower molecular mass of the subcomplex, this indicated a reduction of the turnover number of the subcomplex to 34%. This fitted well to the values determined for mitochondrial membranes (Table S1) and indicated that subcomplex *nb8m*Δ had remained stable during the purification procedure. To test whether the inhibitor sensitive ubiquinone reductase activity of subcomplex *nb8m*Δ was still coupled to proton pumping, we reconstituted it into proteoliposomes and monitored the ΔpH dependent fluorescence quench of 9-amino-6-chloro-2-methoxyacridine (ACMA) as previously described [21]. As shown in Figure 5a, addition of NADH and the ubiquinone analogue DBQ to subcomplex *nb8m*Δ proteoliposomes resulted in the formation of a pH gradient. However, reflecting the lower ubiquinone reductase activity of only 38%, the rate by which it was reached was significantly lower than for holo-complex I. The gradient was maintained at a steady-state plateau over several minutes and was abolished by the addition of the specific inhibitor 2-n-decyl-4-quinazolinyl-amine (DQA) (Figure 5a) or the uncoupler carbonyl-cyanide-*p*-trifluoro-methoxy-phenylhydrazone (FCCP) (Figure S3). This indicated that even the purified subcomplex *nb8m*Δ had retained proton pumping activity, although at a reduced rate. To determine whether the reduced rate of proton pumping by subcomplex *nb8m*Δ was just due to its reduced electron transfer activity or also reflected a lower pumping stoichiometry, we went on to determine the pumping efficiencies of reconstituted parental complex I and subcomplex *nb8m*Δ. When the ACMA fluorescence quench reaches a steady-state plateau, the rates of proton pumping and backflow are equivalent [21]. This fluorescence level should thus reflect the pump rate, provided that the proton leak is not changed. Indeed we found that the rates of proton backflow that can be fitted by a first order exponential function after the addition of DQA (Figure S5) were very similar for proteoliposomes with parental enzyme and with subcomplex *nb8m*Δ (Figure 5b). Also the maximal fluorescence %FL_max_ was essentially identical for the enzymes from both strains (Figure 5b). Importantly, the decrease in fluorescence turned out to be proportional to electron transfer activity up to the point where a complete quench of the ACMA fluorescence was observed (Figure 5c; Figure S4). We concluded that the slope of this plot can be used as a measure for the pumping stoichiometry of complex I. The ACMA fluorescence quench signal is difficult to calibrate in absolute terms, however the pumping stoichiometry for *Y. lipolytica* complex I was previously determined at 4 H^+^/2 e^−^ [22]. Thus the observation that for subcomplex *nb8m*Δ proteoliposomes the slope of the %FL_max_ over activity plot (Figure 5c) was about half of that for parental complex I indicated that the absence of the P_D_ module resulted in a reduced pumping stoichiometry of 2 H^+^/2e^−^.

### Conclusion

**Figure 6:**
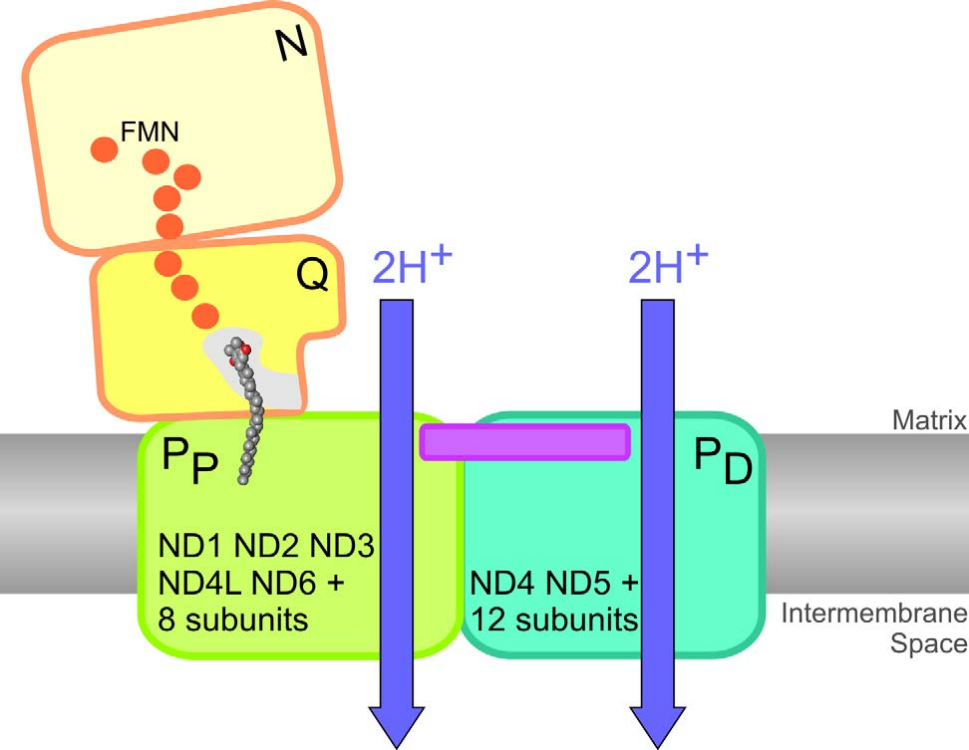
Schematic model of complex I and its pumping modules. Our data are consistent with the dissection of complex I into four functional modules [6] and allow the assignment of subunits and proton pumps to the proximal (P_P_, green) and distal (P_D_, cyan) domains of the membrane arm. The FMN containing N-module (light yellow), the Q-module harbouring the ubiquinone binding site (dark yellow), the chain of seven iron-sulfur clusters (orange spheres), and the extended transmission element (magenta) are indicated.

We concluded that we have functionally dissected two pumping modules of complex I and that these modules correspond to the P_P_ and P_D_ domains of the membrane arm previously identified by X-ray structural analysis (Figure 6; [6]). The fact that the pump in the P_P_ module was still functional in subcomplex *nb8m*Δ provides experimental support for the proposal that the P_D_ domain of complex I indeed harbours a proton pump connected in series to the primary pump in the P_P_ domain. This is consistent with the idea that the long helical transmission element bridging the two subdomains is involved in operating the proton pumps of complex I [5],[6].

## Materials and Methods

### Materials

Asolectin (total soy bean extract with 20% lecithin) was purchased from Avanti Polar Lipids (Alabaster, AL), *n*-dodecyl-ß-D-maltoside from Glycon (Luckenwalde, Germany), and octyl-ß-D-glucopyranoside was from Biomol (Hamburg, Germany). 9-amino-6-chloro-2-methoxyacridine (ACMA) was obtained from Invitogen/Molecular Probes (Eugene, OR), decylubiquinone (DBQ) from Alexis Biochemicals (Lausen, Switzerland). DQA (2-n-decyl-quinazolin-4-yl-amine, SAN 549) was from AgrEvo (Frankfurt, Germany). Carbonyl-cyanide-*p*-trifluoro-methoxy-phenylhydrazone (FCCP), the potassium ionophore valinomycin, and all other chemicals were from Sigma. ACMA, DBQ, DQA, and valinomycin were dissolved in dimethylsulfoxide.

### Construction of Deletion Strain nb8mΔ of *Yarrowia lipolytica*

The *Y. lipolytica* deletion strain *nb8m*Δ (*NUGM-Htg2*, *ndh2i*, *lys11* ^−^, *leu2* ^−^, *ura3* ^−^, *nb8m*::*URA3*, *MatB*), in which a 976 bp fragment corresponding to the complete *NB8M* gene including the intron and up- and downstream sequences was replaced by the *Y. lipolytica URA3* gene (1.6 kb) oriented in opposite direction to the original *NB8M* open reading frame, was constructed by the double homologous recombination strategy as previously published [23].

### Purification of Complex I from Mitochondrial Membranes of *Y. lipolytica*

*Y. lipolytica* strains PIPO and strain *nb8m*Δ were grown in modified YPD medium (2.5% glucose, 2% bactopeptone, 1% yeast extract). Both strains contain a chromosomal copy of the modified *NUGM* gene, encoding a C-terminally his-tagged version of the 30-kDa subunit of complex I [23]. Mitochondrial membranes were prepared following the protocol of [24] with the modification detailed in [18]. Purification of *n*-dodecyl-ß-D-maltoside solubilized complex I was achieved by Ni-affinity chromatography, followed by gel filtration as detailed in [18]. The yield of each preparation was determined by measuring the NADH:HAR oxidoreductase activity while the specific NADH:DBQ oxidoreductase activity was measured after lipid-activation as described [20].

### Electrophoretic and Associated Techniques

Mitochondrial membranes from *Y. lipolytica* were solubilized by 1.5% laurylmaltoside and separated by two-dimensional blue-native electrophoresis (2D BN-PAGE) as described [17]. For the analysis of the subunit composition of the purified *nb8m*Δ subcomplex, dSDS-PAGE [19]) was applied. In-gel catalytic activity stain was performed with solubilized membranes after BN-PAGE as detailed previously [17]. For Western blot analysis of mitochondrial membranes, proteins were transferred to polyvinylidendifluoride membranes and incubated with a monoclonal antibody against the 49-kDa subunit [10]. The secondary antibody was a peroxidase conjugated rabbit anti-mouse IgG and the signal was detected by enhanced chemiluminescence.

### Mass Spectrometry

Laser Induced Liquid Bead Ion Desorption Mass Spectrometry (LILBID-MS) was done as described previously [25]. Proteins from gel-spots were identified by nano-ESI-LC-MS/MS on a Thermo Scientific LTQ Orbitrap XL mass spectrometer essentially as described in [26] except that database searches were done against an in-house compiled version of the *Y. lipolytica* protein database of the Genolevures consortium [27] containing annotations of all known complex I subunits.

### Electron Microscopy

For electron microscopy the subcomplex was prepared on holey carbon coated grids and embedded in stain (NanoW, Nanoprobes, Yaphank, NY), following the deep staining methods described in [28],[29]. For single particle 3D reconstruction using the method of Random Conical Tilting [30], 100 tilt pairs were recorded at a nominal magnification of 67 k×, and a tilt angle of 55°. 68 tilt pairs were used for the reconstruction. The images were scanned with an Intergraph SCAI flatbed scanner (Z/I Imaging Corporation, Huntsville, AL) and reduced to a calibrated pixel size of 3.136 Å. Particle pairs were picked from the 0°- and tilt-micrograph. The microscope contrast transfer function was determined according to Radermacher et al. [31], and all images were corrected by smooth phase flipping. A total of 10,897 image pairs were processed, using a sequence of reference free alignment followed by several rounds of correspondence analysis, followed by classification and multi-reference alignment. The last step in the processing of the 0° images was a classification after correspondence analysis using Diday's method of moving centers and hierarchical ascendant classification [32]. The processing yielded 10 classes of subcomplex volumes that mostly differed in their orientation to the supporting carbon and could be combined into a single reconstruction. The final volume was calculated from 10,586 images. The resolution was determined using the Fourier Shell Correlation with a cutoff of 0.3 [33]. The subcomplex structure was superimposed on the holocomplex from earlier studies [8]. The handedness of all volume representations of complex I has been adjusted to the handedness presented in [5].

### Reconstitution of Complex I into Proteoliposomes

Parental complex I and the subcomplex purified from strain *nb8m*Δ were reconstituted into proteoliposomes, generally following the protocol described in [21] with the modifications detailed in Dröse et al. [34]. 10 mg/ml asolectin solubilized in 16 mg/ml octylglucoside (resulting in “total solubilization” of lipids) were mixed at a protein-to-lipid ratio of 1∶50 (w/w) with purified complex I (0.8–1.0 mg per experimental set) in 20 mM K^+^/Mops pH 7.2, 80 mM KCl and the detergents were removed by BioBeads. Note that due to the reduced molecular mass of the subcomplex, the corresponding molar protein-to-lipid ratio was somewhat lower for subcomplex *nb8m*Δ (i.e., less phospholipids per complex I). Usually, subcomplex *nb8m*Δ and parental complex I were reconstituted and analyzed in parallel on the same day.

### Proton Transport and Activity Determination of the Reconstituted Enzyme

Proteoliposomes containing ∼30 µg of complex I were added to 2 ml buffer (20 mM K^+^/Mops pH 7.2, 80 mM KCl, 0.5 µM valinomycin) in a stirred cuvette. H^+^-translocation was monitored as fluorescence change of ACMA that was added to a final concentration of 0.5 µM. Measurements were performed in a Shimadzu RF-5001 fluorescence spectrophotometer at 30°C (λ_ex_ = 430 nm, λ_em_ = 475 nm, band pass 5 nm, integration time 0.2 s). To gradually reduce the catalytic activity of the applied reconstituted complex I, increasing concentrations of the ubiquinone binding site inhibitor DQA were added prior to the start of the experiment. Each measurement of one dataset followed a strict time protocol. After starting the recording, ACMA was added after 10 s to determine the somewhat variable background fluorescence. At 30 s, 100 µM DBQ were added which caused significant fluorescence quenching due to spectral overlap [21]. This interference stabilized after ∼45 s, and at 75 s 100 µM NADH were added to start complex I driven proton-pumping. At 285 s, this activity was stopped completely by adding DQA to a final concentration of 10 µM. The measurement was terminated after 6 min to allow for backflow analysis (see below). To determine the catalytic activity of the reconstituted complex I, NADH-oxidation rates were recorded using a Shimadzu Multi Spec-1501 diode array spectrophotometer (ε_340–400 nm_ = 6.1 mM^−1^ cm^−1^) under the same experimental conditions and time regime except that the total volume was reduced to 1 ml.

### Data Analysis and Determination of H^+^/e^−^ Stoichiometry

Relative proton pumping rates were determined indirectly from the fluorescence level of the ACMA quench assay assuming that during this steady-state the pump and the leak rates should be equivalent. This approach was found to be valid since the average haftimes of proton backflow were very similar (Figure 5b). We had also considered determining the rate of proton pumping in a more direct way from the initial ACMA quench rate. However, this turned out to be not feasible, since complex I requires a few turnovers for its transition to full activity from its deactive state [21].

To minimize variations from the background fluorescence, the mean value of 48–52 data points between start of the measurement and the addition of ACMA was subtracted. As the starting (maximal) fluorescence value, the average of 11 data points before the addition of NADH (and after settling of the DBQ quench effect) was then calculated and set to 100%. Obvious “outliers” were excluded from these calculations. The fluorescence [%FL_max_] reached at the steady state maximal quench plateau representing the equilibration between the complex I catalyzed H^+^-pumping and the ΔpH-enforced H^+^-leak [21] was determined as the mean value of at least 60 data points and plotted against the NADH:DBQ oxidoreductase activity of the reconstituted enzyme measured in parallel under the same conditions (see above). For complex I from the parental strain data above an activity of ∼1.3 µmol min^−1^ mg^−1^ could not be plotted, since ACMA fluorescence was quenched completely. The activities were corrected for the small residual activity after the final addition of a saturating amount of DQA. Finally the data were fitted by linear regression analysis.

## Supporting Information

Figure S1 Blue native electrophoresis, in-gel activity staining, and Western blot analysis of mitochondrial membranes from strain *nb8m*Δ. Mitochondrial membranes of the parental strain and the deletion strain *nb8m*Δ were solubilized with dodecylmaltoside (1.5 g/g) and separated by BN-PAGE. Two lanes of the BN-gel were subsequently used for the NADH dehydrogenase activity staining assay and two lanes were subjected to Western blot analysis with a monoclonal antibody directed against the 49-kDa subunit. The positions of respiratory chain complexes (I, V, III, and IV) and the subcomplex *nb8m*Δ (I*) are indicated. #, unidentified band with NADH dehydrogenase activity that has been previously observed in *Y. lipolytica* membranes^17^.
(TIF)

Figure S2 LILBID mass fingerprint spectra of complex I and subcomplex *nb8m*Δ. The LILBID anion mass spectra reveal individual subunits of purified holo-complex I and subcomplex *nb8m*Δ. In the range of 6–8 m/z the cation spectra are also shown in red. The 42 known subunits are numbered as in Table 1 and the masses are indicated with vertical lines. Subunits that were absent in subcomplex *nb8m*Δ are marked by “X”. Peaks corresponding to doubly charged subunits are also assigned. ⧫, unidentified contaminating protein; *, unidentified possible 43^rd^ subunit.
(TIF)

Figure S3 Proton pumping of reconstituted complex I and subcomplex *nb8m*Δ. The proton pumping activity was monitored by ACMA quenching. Additions of 0.5 µM ACMA, substrates (60 µM DBQ, 100 µM NADH), inhibitor (10 µM DQA), and uncoupler (1 µM FCCP) are indicated. Note that these control measurements were performed at a lower DBQ concentration resulting in somewhat lower electron transfer activities and a reduced extent of fluorescence quenching for subcomplex *nb8m*Δ. Left panels, holo-complex I; right panels, subcomplex *nb8m*Δ.
(TIF)

Figure S4 Quantification of proton pumping efficiencies by inhibitor titration. Representative dataset that was included in the analysis shown in Figure 5c. The figure shows the quality of the original data. Measurements were started in the presence of the indicated DQA concentrations (0–10 µM) to gradually reduce the activities of the reconstituted holo-complex I and subcomplex *nb8m*Δ. Note that somewhat different DQA concentrations had to be added to the holo-complex I (upper panel) and subcomplex *nb8m*Δ (lower panel) to achieve distribution of quench levels over the entire range. After starting the experiments 0.5 µM ACMA, 100 µM DBQ, 100 µM NADH, and finally DQA to a total concentration of 10 µM (e.g., if 1 µM was present at the start, 9 µM had to be applied) were subsequently added. The NADH:DBQ oxidoreductase activity was monitored in parallel under identical experimental conditions (not shown) and only data within the linear range of the activity/quench dependence were included in the analysis.
(TIF)

Figure S5Determination of the H^+^-leak of proteoliposomes with reconstituted holocomplex I and subcomplex *nb8m*Δ. After addition of the inhibitor DQA to a final concentration of 10 µM, the ACMA fluorescence increased exponentially due to the passive backflow of protons (Figure S4). To obtain the halftime τ of the proton leak, the backflow curves were fitted using the Origin 6.0 software package by the function:
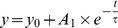
Only data above ∼70% FL_max_ were included in the fit to remove the mixing artefact that also prevented fitting signals returning from a smaller maximal quench amplitude. Representative analysis of two measurements from Figure S4 is shown. The half times (τ) obtained for the shown examples were τ = 15.0 s for holo-complex I (R^2^ = 0.992) and τ = 13.6 s for subcomplex *nb8m*Δ (R^2^ = 0.994).(TIF)

Table S1 NADH:HAR oxidoreductase and inhibitor sensitive dNADH:DBQ oxidoreductase activities of mitochondrial membranes.
(PDF)

## Abbreviations

2D BN/SDS-PAGE: blue-native polyacrylamide electrophoresis
ACMA: 9-amino-6-chloro-2-methoxyacridine
DBQ: decylubiquinone
(d)NADH: (deamino) nicotinamide adenine dinucleotide
DQA: 2-n-decyl-4-quinazolinyl-amine
dSDS-PAGE: doubled sodium dodecyl sulfate polyacrylamide electrophoresis
ESI: electrospray ionisation
FCCP: carbonyl-cyanide-*p*-trifluoro-methoxy-phenylhydrazone
HAR: hexammineruthenium
LILBID: laser induced liquid bead ion desorption
MS: mass spectrometry
