# Supplementary figures and images for: Functional Dissection of the Proton Pumping Modules of Mitochondrial Complex I
Source: PLoS Biol. 2011 Aug 23;9(8):e1001128. doi: 10.1371/journal.pbio.1001128 (PMC3160329; doi:10.1371/journal.pbio.1001128)

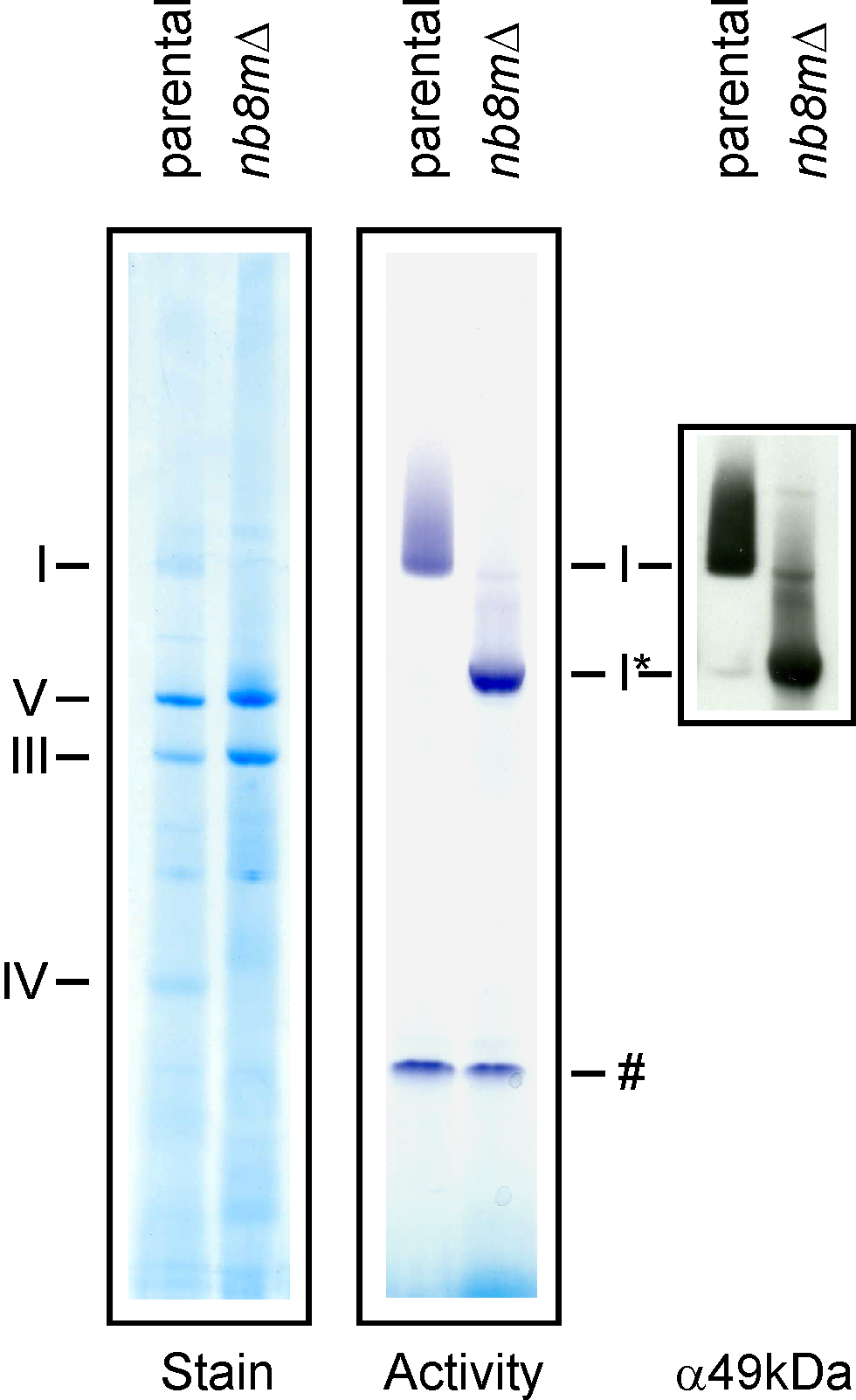

Supplement: Figure S1 — Blue native electrophoresis, in-gel activity staining, and Western blot analysis of mitochondrial membranes from strain nb8mΔ. Mitochondrial membranes of the parental strain and the deletion strain nb8mΔ were solubilized with dodecylmaltoside (1.5 g/g) and separated by BN-PAGE. Two lanes of the BN-gel were subsequently used for the NADH dehydrogenase activity staining assay and two lanes were subjected to Western blot analysis with a monoclonal antibody directed against the 49-kDa subunit. The positions of respiratory chain complexes (I, V, III, and IV) and the subcomplex nb8mΔ (I*) are indicated. #, unidentified band with NADH dehydrogenase activity that has been previously observed in Y. lipolytica membranes17. (TIF) [file pbio.1001128.s001.tif]

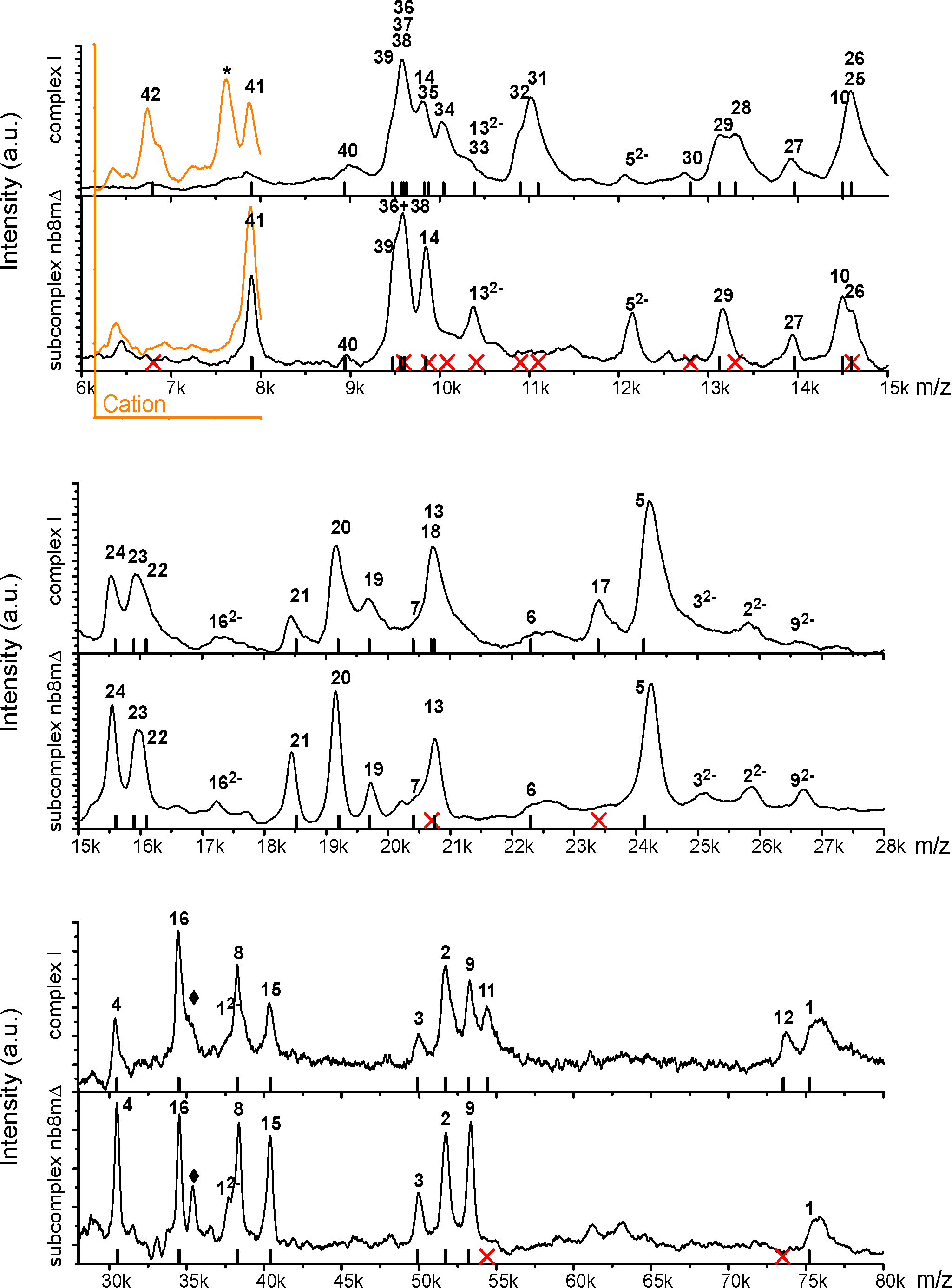

Supplement: Figure S2 — LILBID mass fingerprint spectra of complex I and subcomplex nb8mΔ. The LILBID anion mass spectra reveal individual subunits of purified holo-complex I and subcomplex nb8mΔ. In the range of 6–8 m/z the cation spectra are also shown in red. The 42 known subunits are numbered as in Table 1 and the masses are indicated with vertical lines. Subunits that were absent in subcomplex nb8mΔ are marked by “X”. Peaks corresponding to doubly charged subunits are also assigned. ⧫, unidentified contaminating protein; *, unidentified possible 43rd subunit. (TIF) [file pbio.1001128.s002.tif]

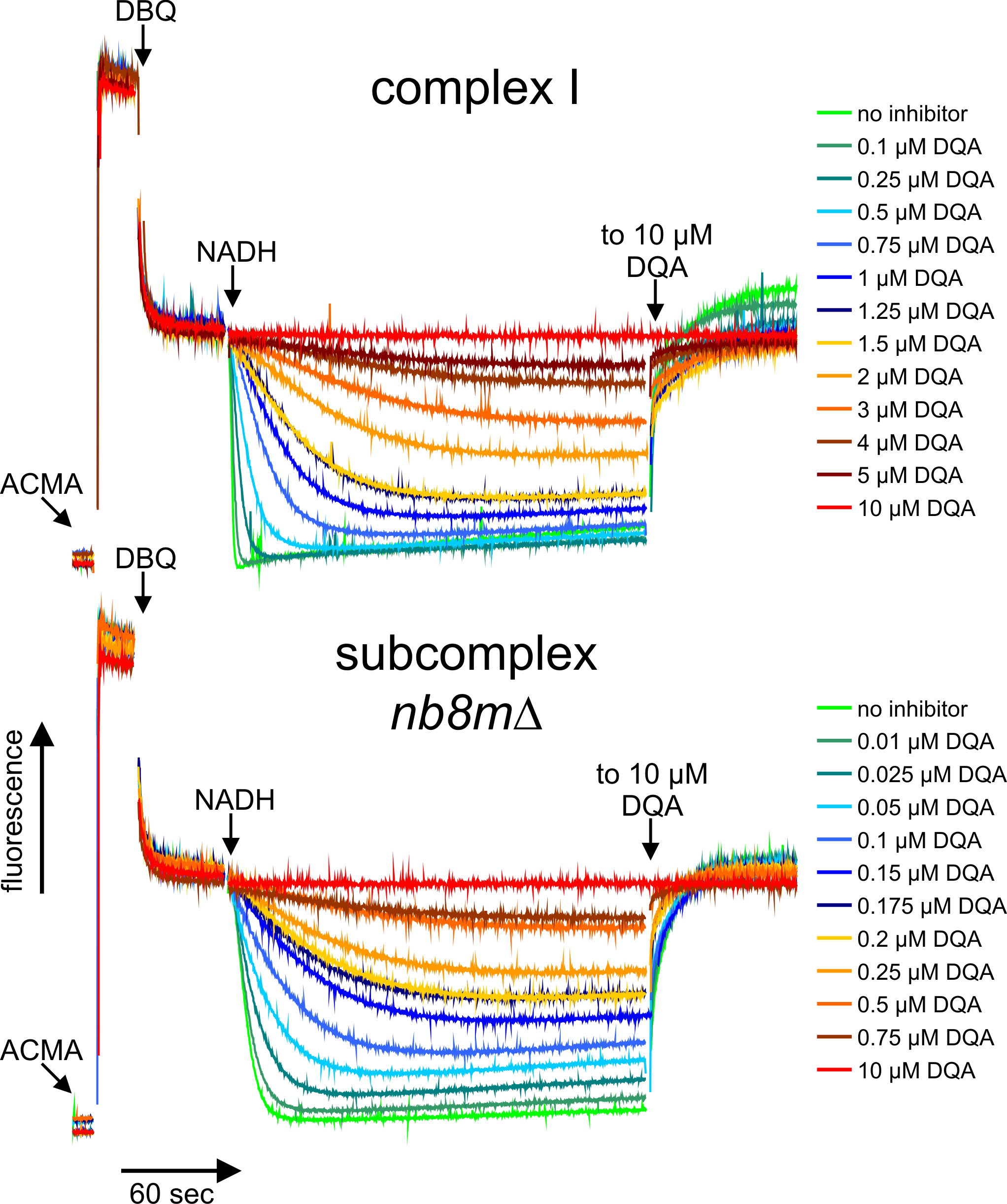

Supplement: Figure S4 — Quantification of proton pumping efficiencies by inhibitor titration. Representative dataset that was included in the analysis shown in Figure 5c. The figure shows the quality of the original data. Measurements were started in the presence of the indicated DQA concentrations (0–10 µM) to gradually reduce the activities of the reconstituted holo-complex I and subcomplex nb8mΔ. Note that somewhat different DQA concentrations had to be added to the holo-complex I (upper panel) and subcomplex nb8mΔ (lower panel) to achieve distribution of quench levels over the entire range. After starting the experiments 0.5 µM ACMA, 100 µM DBQ, 100 µM NADH, and finally DQA to a total concentration of 10 µM (e.g., if 1 µM was present at the start, 9 µM had to be applied) were subsequently added. The NADH:DBQ oxidoreductase activity was monitored in parallel under identical experimental conditions (not shown) and only data within the linear range of the activity/quench dependence were included in the analysis. (TIF) [file pbio.1001128.s004.tif]

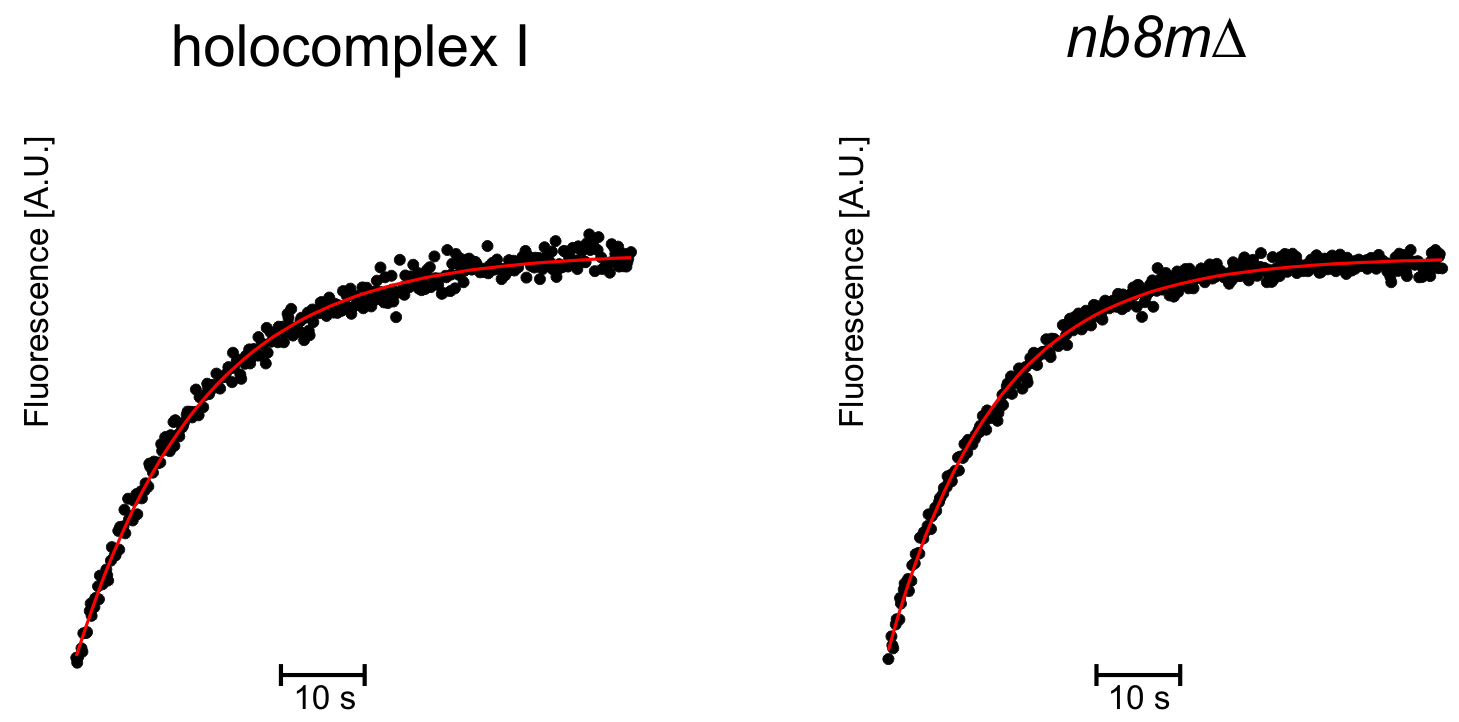

Supplement: Figure S5 — Determination of the H+-leak of proteoliposomes with reconstituted holocomplex I and subcomplex nb8mΔ. After addition of the inhibitor DQA to a final concentration of 10 µM, the ACMA fluorescence increased exponentially due to the passive backflow of protons (Figure S4). To obtain the halftime τ of the proton leak, the backflow curves were fitted using the Origin 6.0 software package by the function:Only data above ∼70% FLmax were included in the fit to remove the mixing artefact that also prevented fitting signals returning from a smaller maximal quench amplitude. Representative analysis of two measurements from Figure S4 is shown. The half times (τ) obtained for the shown examples were τ = 15.0 s for holo-complex I (R2 = 0.992) and τ = 13.6 s for subcomplex nb8mΔ (R2 = 0.994). (TIF) [file pbio.1001128.s005.tif]
