# Supplementary material for: Functional Dissection of the Proton Pumping Modules of Mitochondrial Complex I
Source: PLoS Biol. 2011 Aug 23;9(8):e1001128. doi: 10.1371/journal.pbio.1001128 (PMC3160329; doi:10.1371/journal.pbio.1001128)
Supplement: Table S1 — NADH:HAR oxidoreductase and inhibitor sensitive dNADH:DBQ oxidoreductase activities of mitochondrial membranes. (PDF) [file pbio.1001128.s006.pdf]

Table S1: *NADH:HAR oxidoreductase and inhibitor sensitive dNADH:DBQ oxidoreductase activities of mitochondrial membranes.*

Strain	NADH:HAR oxidoreductase activity		dNADH:DBQ oxidoreductase activity	
	µmol min^{-1} mg^{-1}	%	µmol min^{-1} mg^{-1}	%
parental	0.96	100	0.53	100
*nb8m*Δ	1.04	108	0.16	30
